# Delirium at the elderly patients with alcohol withdrawal syndrome

**DOI:** 10.1192/j.eurpsy.2021.1125

**Published:** 2021-08-13

**Authors:** V. Kuzminov, I. Linskiy

**Affiliations:** Department Of Emergency Psychiatry And Narcology, SI Institute of Neurology, Psychiatry and Narcology NAMS of Ukraine, Kharkiv, Ukraine

**Keywords:** alcohol withdrawal syndrome, delirium, elderly patients

## Abstract

**Introduction:**

The psychoses in patients with dependence of alcohol are in many cases polyethiologic, this is especially common in the elderly due to accumulation of various somato-neurological pathologies.

**Objectives:**

88 men, with alcohol withdrawal syndrome accompanied by delirium; the average age - 70,4 ± 3,9 years, duration of alcohol abuse - 27,4 ± 6,5 years.

**Methods:**

clinical, psychopathological and statistical

**Results:**

The psychoses in patients with dependence of alcohol are in many cases polyethiologic, this is especially common in elderly due to accumulation of various somato-neurological pathologies. One of such factors is alcohol dependence syndrome and alcohol withdrawal. ICD-10 allows sharing out delirium with mixed etiology F05.8; this category can include patients when there is a severe alcohol withdrawal condition and somato-neurological pathology that can be an independent factor in the delirious syndrome. 88 elderly patients with were examined in state of alcohol withdrawal. All patients had delirious syndrome. Patients were divided into 2 groups: 1st – patients with a condition of alcohol withdrawal with delirium; 2nd - patients with a delirium of mixed etiology (the factor of the presence of dyscirculatory encephalopathy, was considered a competing factor in the onset of delirium). Some differential-diagnostic signs of the studied disorders were established. In the case of prolongation of psychosis, the clinical picture was similar in both groups, which was explained by exacerbation of the existing somatic pathology.

**Conclusions:**

Estimation of the leading factor in the emergency of acute psychosis in patients with alcohol withdrawal syndrome is of great practical importance for selection of therapeutic tactics.

